# Triglyceride-glucose index in the prediction of adverse cardiovascular events in patients without diabetes mellitus after coronary artery bypass grafting: a multicenter retrospective cohort study

**DOI:** 10.1186/s12933-023-01969-3

**Published:** 2023-08-30

**Authors:** Zhenguo Wu, Lin Xie, Dachuan Guo, Sha Chen, Xiaoyu Liu, Xiangfei Sun, Juan Wang, Yerui Zhang, Li Liu, Huiliang Cui, Dejin Zang, Jianmin Yang

**Affiliations:** 1 National Key Laboratory for Innovation and Transformation of Luobing Theory, The Key Laboratory of Cardiovascular Remodeling and Function Research, Chinese Ministry of Education, Chinese National Health Commission and Chinese Academy of Medical Sciences, Department of Cardiology, Qilu Hospital of Shandong University, Jinan, China; 2 Department of Cardiovascular Surgery, Shandong Provincial Hospital, Cheeloo College of Medicine, Shandong University, Jinan, Shandong China; 3 Department of Cardiovascular Surgery, Shandong Provincial Hospital Affiliated to Shandong First Medical University, Jinan, Shandong China; 4 Department of Cardiology, The Second Hospital of Shandong University, Jinan, Shandong China

**Keywords:** Triglyceride-glucose index, Coronary artery bypass grafting, Insulin resistance, Prognosis, Cohort study

## Abstract

**Background:**

The triglyceride-glucose (TyG) index has been evaluated as a reliable surrogate for insulin resistance (IR) and has been proven to be a predictor of poor outcomes in patients with cardiovascular diseases. However, data are lacking on the relationship of the TyG index with prognosis in nondiabetic patients who underwent coronary artery bypass grafting (CABG). Thus, the purpose of our current study was to investigate the potential value of the TyG index as a prognostic indicator in patients without diabetes mellitus (DM) after CABG.

**Methods:**

This multicenter, retrospective cohort study involving 830 nondiabetic patients after CABG from 3 tertiary public hospitals from 2014 to 2018. Kaplan–Meier survival curve analysis was conducted followed by the log-rank test. Cox proportional hazards regression models were used to explore the association between the TyG index and major adverse cardiovascular events (MACEs). The incremental predictive power of the TyG index was evaluated with C-statistics, continuous net reclassification improvement (NRI) and integrated discrimination improvement (IDI).

**Results:**

An incrementally higher TyG index was associated with an increasingly higher cumulative incidence of MACEs (log-rank test, *p* < 0.001). The hazard ratio (95% CI) of MACEs was 2.22 (1.46–3.38) in tertile 3 of the TyG index and 1.38 (1.18–1.62) per SD increase in the TyG index. The addition of the TyG index yielded a significant improvement in the global performance of the baseline model [C-statistic increased from 0.656 to 0.680, *p* < 0.001; continuous NRI (95% CI) 0.269 (0.100–0.438), *p* = 0.002; IDI (95% CI) 0.014 (0.003–0.025), *p* = 0.014].

**Conclusions:**

The TyG index may be an independent factor for predicting adverse cardiovascular events in nondiabetic patients after CABG.

**Supplementary Information:**

The online version contains supplementary material available at 10.1186/s12933-023-01969-3.

## Background

Despite ongoing advances in the prevention and treatment of atherosclerosis, coronary artery disease (CAD) remains one of the leading causes of morbidity and death worldwide [1, 2]. Coronary artery bypass grafting (CABG) is an effective treatment for CAD and is the preferred revascularization strategy for patients with severe multivessel disease [3]. Although advances in surgical techniques have enhanced the efficacy and safety of CABG, the long-term prognosis after CABG remains poor [4, 5].

Insulin resistance (IR), which is a prominent characteristic of metabolic syndrome and diabetes mellitus (DM), also contributes to the acceleration of atherosclerosis through proinflammatory and prothrombotic features [6–8]. Several studies have shown that IR negatively affects the outcomes of myocardial revascularization [9–11]. These findings reveal that early identification of IR has clinical implications in the prevention of adverse events after CABG.

The triglyceride-glucose (TyG) index, a product of triglycerides and glucose, has been evaluated as a reliable surrogate for IR and demonstrated a high concordance with the hyperinsulinemic-euglycemic clamp [12–14]. Previous studies showed that the TyG index was associated with multiple cardiovascular risk factors, such as diabetes, hypertension, metabolic syndrome, arterial stiffness and coronary artery calcification [15–20]. A high TyG index was also shown to predict poor outcomes in patients with CAD [21, 22]. However, studies concerning the clinical utility of the TyG index for CABG were limited to patients with DM [23, 24]. Thus, we conducted the present research to explore whether the TyG index could be used as a prognostic indicator in nondiabetic patients after CABG.

## Methods

### Study design and patients

The study was approved by the Ethics Review Committee of Shandong Provincial Hospital, Qilu Hospital of Shandong University and The Second Hospital of Shandong University, and was performed according to the Declaration of Helsinki. The ethics committee permitted verbal consent because of the retrospective design of this study and the phone follow-up.

**Fig. 1:**
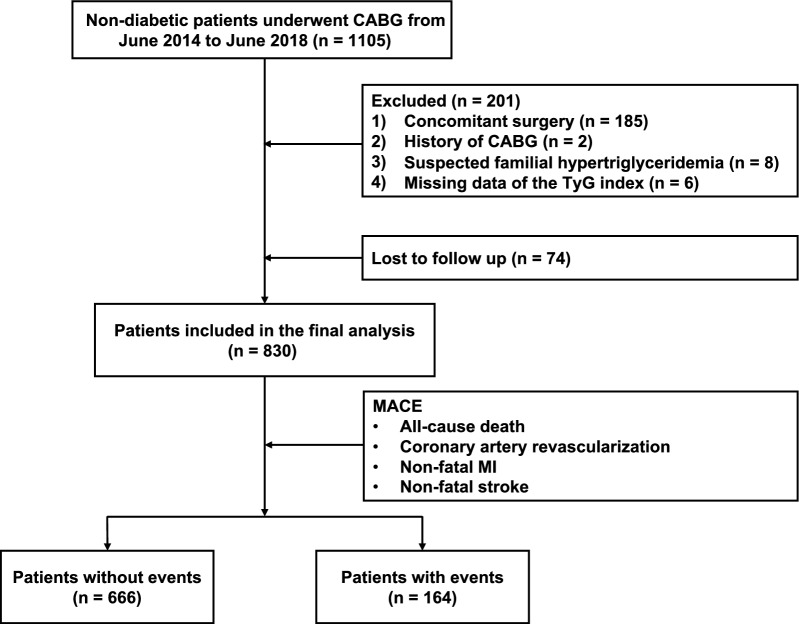
Flow diagram of patient selection. *CABG* coronary artery bypass grafting, *MACE* major adverse cardiovascular events, *MI* myocardial infarction

This study was a multicenter, observational, retrospective cohort study and was conducted at 3 tertiary public hospitals. Nondiabetic patients who underwent isolated CABG for the first time from June 2014 to June 2018 at Qilu Hospital of Shandong University, Shandong Provincial Hospital and The Second Hospital of Shandong University were reviewed retrospectively. Diabetes was defined as fasting plasma glucose (FPG) ≥ 7.0 mmol/L, 2-h plasma glucose after oral glucose tolerance test (OGTT) ≥ 11.1 mmol/L, random blood glucose ≥ 11.1 mmol/L, glycated hemoglobin (HbA1c) ≥ 6.5%, or self-reported history of diabetes which was confirmed by review of corresponding medical records. Patients who underwent concomitant surgery such as valve surgery, surgical ablation or congenital heart surgery were excluded. Those with a history of CABG, suspected familial hypertriglyceridemia (triglyceride ≥ 5.65 mmol/L), or missing data for the TyG index calculation were also excluded. In total, 904 patients were enrolled. The telephone follow-up was conducted from July 2022 to September 2022. Finally, 830 (91.8%) participants provided verbal consent and completed the full questionnaires (Fig. 1).

### Data collection

Clinical data were collected from the electronic medical recording system. The data included patients’ general conditions [age, sex, weight, height, left ventricular ejection fraction (LVEF), medical history and extent of CAD], risk factors [family history of CAD (FH-CAD), smoking, drinking, hypertension, hyperlipidemia], surgical procedure [duration of surgery, use of cardiopulmonary bypass, number of grafts, use of arterial grafts, complete revascularization and use of intra-aortic balloon pump (IABP)], laboratory indicators [FPG, lipid profile and serum creatinine (SCr)], and cardiovascular medication information [antiplatelet drugs, statins, angiotensin-converting enzyme inhibitors (ACEIs)/angiotensin receptor blockers (ARBs), beta-blockers and diuretics]. Current drinking was defined as having at least 1 alcoholic beverage per week in the 12 months before admission and still drinking in this manner at the time of admission. Fasting elbow venous blood samples were collected between 7:00–9:00 a.m. Clinical symptoms and diagnostic changes on electrocardiogram or elevated cardiac biomarkers were used together to confirm the incidence of myocardial infarction (MI) [25]. Patients with at least one first-degree relative with CAD (men < 55, women < 65 years old) were considered to have FH-CAD. Patients with ≥ 50% stenosis in  ≥ 2 major coronary arteries were identified as having multivessel disease and those with ≥ 50% stenosis in the left main coronary artery were identified as containing left main disease. Hypertension was diagnosed according to the following criteria: systolic blood pressure ≥ 140 mmHg and/or diastolic blood pressure ≥ 90 mmHg. Patients who received antihypertensive treatment were also identified as having hypertension in the current study. ICD-10 code E78 was used to define hyperlipidemia [26]. We used SCr to calculate the estimated glomerular filtration rate (eGFR) according to the Chronic Kidney Disease Epidemiology Collaboration (CKD-EPI) equation [27]. The TyG index was determined using the following formula: Ln [fasting triglyceride (TG) (mg/dL) × FPG (mg/dL)/2] [28].

### Endpoint definition

In the current study, the primary observational endpoint was the composite of all-cause death, nonfatal MI, nonfatal stroke and coronary artery revascularization [(major adverse cardiovascular events (MACEs)]. All-cause death referred to death resulting from any cause, including cardiac or noncardiac death. Coronary artery revascularization was defined as any unplanned revascularization for ischemia. Secondary endpoints were defined as the occurrence of each of these components separately. Only the first event was used for analysis for patients with more than one event.

### Statistical analysis

Statistical analysis was performed using SPSS version 25.0 (SPSS, Chicago, IL, United States) and R software version 4.1.3 (R Foundation for Statistical Computing, Vienna, Austria). A *p* value of less than 0.05 was considered to indicate statistical significance. Categorical variables are expressed as numbers (percentage) when describing the baseline characteristics and continuous variables are expressed as the mean ± SD or median (interquartile range). In the comparison of categorical variables, we used the chi-square test. ANOVA was used for normally distributed continuous variables and the Kruskal–Wallis H test was used for skewed continuous variables. Kaplan–Meier survival curve analysis classified by TyG index tertiles was conducted followed by the log-rank test. To identify prognostic predictors in patients after CABG, univariate Cox regression analysis was performed. We used multivariate Cox proportional hazards regression to investigate whether the TyG index could be regarded as an independent risk factor. Covariates were included in models in three stages: Model 1 included age and sex. Variables with *p* < 0.05 in univariate analysis were entered for multivariate analysis in Model 2. All adjustment variables, including age, sex, previous MI, previous stroke, previous PCI, left main disease, multivessel disease, BMI, LVEF, smoking, drinking, hypertension, hyperlipidemia, FH-CAD, duration of surgery, off-pump coronary artery bypass grafting (OPCABG), number of grafts, use of arterial grafts, eGFR, TC, low-density lipoprotein cholesterol (LDL-C), high-density lipoprotein cholesterol (HDL-C), European System for Cardiac Operative Risk Evaluation score II (EuroSCORE II) and medication use, were included in the fully adjusted model (Model 3). The TyG index was included in separate regression equations as both categorical (tertile 1: TyG index < 8.36; tertile 2: 8.36 ≤ TyG index < 8.77; and tertile 3: TyG index ≥ 8.77) variables and continuous variables, and was converted to a z score to determine the increase in the risk of the outcome per SD increase. The variables included in the models were checked for multicollinearity using the variance inflation factor (VIF) values. Given the VIF of < 5, there was no evidence of collinearity among all variables. Schoenfeld residuals were used to test the PH assumption of the Cox regression model and we found that the PH assumption was satisfied (Schoenfeld individual test for each covariate: all *p* values ≥ 0.05, global Schoenfeld test: *p* = 0.943). Subgroup analysis was conducted according to age, sex, BMI, hypertension, and hyperlipidemia. P values for interaction were calculated to explore the effect of each subgroup on the outcome. The receiver operating characteristic (ROC) curves of the regression models at the end of full follow-up were plotted. Model discrimination was evaluated using concordance statistics (C-statistics), which were compared at the end of the follow-up. The risk reclassification was further evaluated using net reclassification improvement (NRI) and integrated discrimination improvement (IDI).

## Results

### Baseline characteristics

A total of 830 nondiabetic patients who underwent CABG served as the final cohort for analysis, consisting of 617 (74.3%) male participants with an average age of 62.79 ± 8.17 years. Table 1 describes the baseline characteristics of the study participants. Age, BMI and lipid profile were significantly different among the three groups. Meanwhile, there were significant differences in the proportion of hypertension, hyperlipidemia and prediabetes. Importantly, more patients had adverse events in the higher TyG index group (Table 1).

**Table 1:** Baseline characteristics of participants according to the tertiles of the TyG index

Variables	Tertile 1 (n = 277)	Tertile 2 (n = 276)	Tertile 3 (n = 277)	*p*-value
TyG index	8.04 ± 0.26	8.57 ± 0.12	9.17 ± 0.33	**< 0.001**
General conditions				
Age (years)	63.57 ± 7.98	62.93 ± 7.88	61.88 ± 8.58	**0.048**
Male, n (%)	218 (78.7)	206 (74.6)	193 (69.7)	0.051
BMI (kg/m^2^)	24.48 ± 3.41	26.00 ± 3.83	26.31 ± 3.51	**< 0.001**
LVEF (%)	58.56 ± 10.30	59.01 ± 9.63	58.86 ± 10.56	0.866
Prediabetes, n (%)	19 (6.9)	55 (19.9)	124 (44.8)	**< 0.001**
Previous MI, n (%)	49 (17.7)	53 (19.2)	68 (24.5)	0.110
Previous stroke, n (%)	33 (11.9)	34 (12.3)	43 (15.5)	0.390
Previous PCI, n (%)	27 (9.7)	29 (10.5)	23 (8.3)	0.669
Left main disease, n (%)	54 (19.5)	61 (22.1)	76 (27.4)	0.077
Multivessel disease, n (%)	257 (92.8)	254 (92.0)	263 (94.9)	0.365
Risk factors, n (%)				
Current smoking	86 (31.0)	71 (25.7)	85 (30.7)	0.306
Current drinking	73 (26.4)	71 (25.7)	80 (28.9)	0.676
FH-CAD	51 (18.4)	45 (16.3)	60 (21.7)	0.267
Hypertension	153 (55.2)	157 (56.9)	183 (66.1)	**0.020**
Hyperlipidemia	66 (23.8)	82 (29.7)	99 (35.7)	**0.009**
Surgical procedure				
Duration of surgery (min)	270.00 (235.00–310.00)	270.00 (230.00–313.75)	270.00 (240.00–325.00)	0.483
OPCABG, n (%)	250 (90.3)	250 (90.6)	239 (86.3)	0.197
Number of grafts	3.53 ± 1.01	3.65 ± 1.04	3.66 ± 0.98	0.259
Use of arterial grafts, n (%)	264 (95.3)	264 (95.7)	266 (96.0)	0.917
Left internal mammary artery, n (%)	248 (89.5)	243 (88.0)	249 (89.9)	0.760
Right internal mammary artery, n (%)	25 (9.0)	30 (10.9)	26 (9.4)	0.741
Radial artery, n (%)	10 (3.6)	12 (4.3)	11 (4.0)	0.906
Complete revascularization, n (%)	260 (93.9)	254 (92.0)	259 (93.5)	0.665
Use of IABP, n (%)	10 (3.6)	12 (4.3)	11 (4.0)	0.906
Laboratory tests				
FPG (mmol/L)	4.66 (4.33–5.07)	5.02 (4.71–5.61)	5.57 (4.98–6.01)	**< 0.001**
TC (mmol/L)	3.85 (3.20–4.50)	4.01 (3.50–4.97)	4.51 (3.83–5.14)	**< 0.001**
TG (mmol/L)	0.87 (0.70–1.01)	1.30 (1.14–1.47)	1.96 (1.61–2.46)	**< 0.001**
LDL-C (mmol/L)	2.20 (1.74–2.76)	2.55 (2.01–3.12)	2.69 (2.12–3.35)	**< 0.001**
HDL-C (mmol/L)	1.19 ± 0.25	1.12 ± 0.29	1.08 ± 0.23	**< 0.001**
eGFR (mL/min/1.73m^2^)	91.35 ± 13.15	90.63 ± 13.45	90.70 ± 13.61	0.787
Cardiovascular medications, n (%)				
Antiplatelet drugs	272 (98.2)	268 (97.1)	272 (98.2)	0.595
Statins	227 (81.9)	237 (85.9)	226 (81.6)	0.329
ACEI/ARB	102 (36.8)	117 (42.4)	121 (43.7)	0.218
Beta-blockers	247 (89.2)	254 (92.0)	242 (87.4)	0.196
Diuretics				
Loop diuretics	24 (8.7)	27 (9.8)	33 (11.9)	0.436
Thiazide diuretics	40 (14.4)	38 (13.8)	48 (17.3)	0.463
Mineralocorticoid receptor antagonists	46 (16.6)	39 (14.1)	50 (18.1)	0.450
EuroSCORE II	1.35 (0.97–2.34)	1.35 (0.99–2.44)	1.38 (0.95–2.10)	0.613
MACE, n (%)	39 (14.1)	45 (16.3)	80 (28.9)	**< 0.001**

*p* values in bold are < 0.05

*TyG index* triglyceride-glucose index, *MI* myocardial infarction, *LVEF* left ventricle ejection fraction, *PCI* percutaneous coronary intervention, *FH-CAD* family history of coronary artery disease, *BMI* body mass index, *OPCABG* off-pump coronary artery bypass grafting, *IABP* intra-aortic balloon pump, *FPG* fasting plasma glucose, *TC* total cholesterol, *TG* triglyceride, *LDL-C* low-density lipoprotein-cholesterol, *HDL-C* high-density lipoprotein-cholesterol, *eGFR* estimated glomerular filtration rate, *ACEI* angiotensin-converting enzyme inhibitors, *ARB* angiotensin receptor blockers, *EuroSCORE* European System for Cardiac Operative Risk Evaluation score, *MACE* major adverse cardiovascular event

### Association between the TyG index and MACEs

**Fig. 2:**
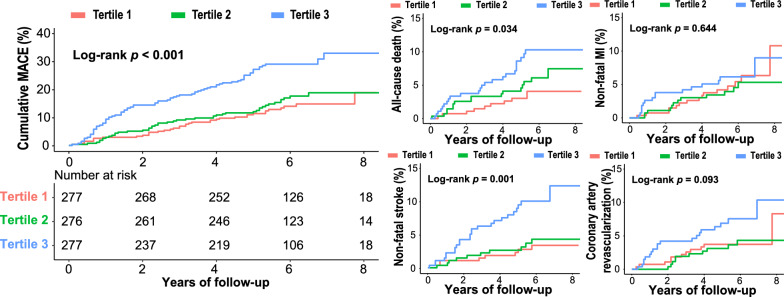
Kaplan–Meier survival curves for the primary and secondary endpoints across the TyG index tertiles. *TyG index* triglyceride-glucose index, *MACE* major adverse cardiovascular events, *MI* myocardial infarction

During a median follow-up of 69 (57–77) months, 164 patients (19.8%) developed at least one endpoint event. Kaplan–Meier survival plots for the incidence of MACEs by the TyG index tertiles are presented in Fig. 2. An incrementally higher TyG index was associated with an increasingly higher cumulative incidence of MACEs (log-rank test, *p* < 0.001). Similar results were observed for the prediction of all-cause death and nonfatal stroke (log-rank test, both *p*-values < 0.05). However, the TyG index could not significantly distinguish the patients with a higher risk of nonfatal MI or coronary artery revascularization (log-rank test, *p* > 0.05) (Fig. 2).

The results of the univariate Cox regression analysis are displayed in Table 2. Age, LVEF, multivessel disease, duration of surgery, FPG, TC, TG, eGFR, EuroSCORE II and the TyG index were significantly associated with MACEs. The unadjusted HR of MACE risk per SD increase in the TyG index was 1.43 (95% CI: 1.23–1.66, *p* < 0.001) (Table 2). In Model 2, the TyG index was a significant factor for poor prognosis. In Model 3, this association remained significant after adjusting for other potential confounders, regardless of whether the TyG index was considered as a categorical or continuous variable. The test for trends across tertiles of the TyG index for the risk of MACEs was statistically significant (Table 3).

**Table 2:** Univariate Cox regression analyses for MACE

Variables	HR	95% CI	*p*-value
Age	1.03	1.01–1.05	**0.001**
Male	1.06	0.74–1.52	0.747
BMI	1.02	0.98–1.06	0.342
LVEF	0.18	0.04–0.75	**0.018**
Previous MI	1.05	0.72–1.52	0.807
Previous stroke	1.18	0.77–1.81	0.439
Previous PCI	0.92	0.54–1.56	0.751
Left main disease	1.17	0.82–1.67	0.378
Multivessel disease	2.48	1.02–6.04	**0.046**
Current smoking	1.20	0.87–1.66	0.272
Current drinking	1.05	0.75–1.48	0.773
FH-CAD	0.96	0.64–1.42	0.827
Hypertension	1.33	0.96–1.83	0.086
Hyperlipidemia	1.19	0.86–1.65	0.303
Duration of surgery	1.00	1.00–1.01	**0.011**
OPCABG	1.43	0.92–2.22	0.115
Number of grafts	0.97	0.83–1.13	0.666
Use of arterial grafts	1.06	0.50–2.26	0.878
FPG	1.19	1.09–1.29	**< 0.001**
TC	1.20	1.08–1.33	**0.001**
TG	1.41	1.19–1.68	**< 0.001**
LDL-C	1.15	0.98–1.35	0.083
HDL-C	1.16	0.64–2.10	0.616
eGFR	0.98	0.97–0.99	**0.001**
Antiplatelet drugs	0.91	0.13–6.46	0.921
Statins	0.89	0.60–1.32	0.549
EuroSCORE II	1.11	1.04–1.19	**0.003**
TyG index	1.97	1.49–2.61	**< 0.001**
TyG index (Per SD)	1.43	1.23–1.66	**< 0.001**

*MACE* major adverse cardiovascular events, *MI* myocardial infarction, *BMI* body mass index, *PCI* percutaneous coronary intervention, *LVEF* left ventricle ejection fraction, *FH-CAD* family history of coronary artery disease, *OPCABG* off-pump coronary artery bypass grafting, *FPG* fasting plasma glucose, *TC* total cholesterol, *TG* triglyceride, *LDL-C* low-density lipoprotein-cholesterol, *HDL-C* high-density lipoprotein-cholesterol, *eGFR* estimated glomerular filtration rate, *EuroSCORE* European System for Cardiac Operative Risk Evaluation score, *TyG index* triglyceride-glucose index, *SD* standard deviation, *HR* Hazard ratio, *CI* Confidence interval

*p* values in bold are < 0.05

**Table 3:** Multivariate Cox regression analysis for MACE

TyG index	HR (95% CI)		
	Model 1	Model 2	Model 3
Per Unit increase	2.07 (1.57–2.74)***	1.84 (1.38–2.46)***	1.85 (1.36–2.50)***
Per SD increase	1.47 (1.27–1.70)***	1.38 (1.19–1.61)***	1.38 (1.18–1.62)***
Tertile 1	1 (Reference)	1 (Reference)	1 (Reference)
Tertile 2	1.21 (0.79–1.86)	1.14 (0.74–1.76)	1.15 (0.73–1.81)
Tertile 3	2.49 (1.69–3.66)***	2.20 (1.49–3.26)***	2.22 (1.46–3.38)***
*p* for trend	**< 0.001**	**< 0.001**	**< 0.001**

^***^ *p* < 0.001

*p* values in bold are < 0.05

In addition, the sensitivity analysis indicated that our results were not materially changed even after excluding noncardiac death, those taking lipid-lowering drugs at admission, or individuals who developed DM during the follow-up (Additional file 1: Table S1).

### Predictive ability of the TyG index for the secondary outcomes

We further studied the associations between the TyG index and all-cause death, coronary artery revascularization, nonfatal MI and nonfatal stroke. Compared with subjects in the lowest tertile, patients in the highest tertile presented a statistically significant increase in risk for all-cause death and nonfatal stroke. No statistical significance was observed when predicting nonfatal MI and coronary artery revascularization (Table 4).

**Table 4:** Multivariate Cox regression analysis for secondary endpoints

TyG index	HR (95% CI)			
	All-cause death	Coronary artery revascularization	Non-fatal MI	Non-fatal stroke
Per Unit increase	1.63 (0.95–2.79)	1.75 (0.91–3.38)	1.43 (0.77–2.67)	2.16 (1.20–3.86)*
Per SD increase	1.29 (0.97–1.72)	1.35 (0.95–1.91)	1.21 (0.87–1.68)	1.50 (1.10–2.04)*
Tertile 1	1 (Reference)	1 (Reference)	1 (Reference)	1 (Reference)
Tertile 2	1.55 (0.68–3.56)	0.84 (0.34–2.05)	0.87 (0.39–1.96)	0.87 (0.34–2.27)
Tertile 3	2.92 (1.29–6.61)*	1.58 (0.69–3.62)	1.30 (0.60–2.81)	2.36 (1.04–5.36)*
*p* for trend	**0.008**	0.231	0.485	**0.016**

Adjusted for age, gender, BMI, LVEF, previous MI, previous stroke, previous PCI, left main disease, multivessel disease, current smoking, current drinking, FH-CAD, hypertension, hyperlipidemia, duration of surgery, OPCABG, number of grafts, use of arterial grafts, TC, LDL-C, HDL-C, eGFR, EuroSCORE II, antiplatelet drugs and statins

^*^ *p* < 0.05

*p* values in bold are < 0.05

### Subgroup analysis

**Fig. 3:**
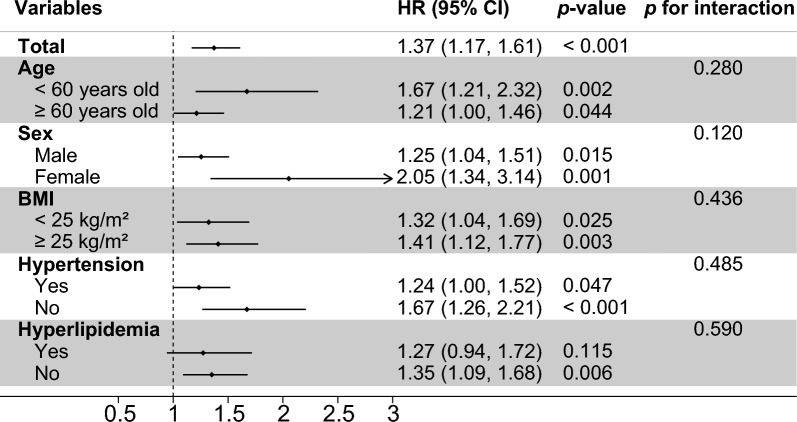
Subgroup and interaction analysis between the TyG index (Per SD) and MACE across various subgroups. *TyG index* triglyceride-glucose index, *MACE* major adverse cardiovascular events, *SD* standard deviation, *HR* Hazard ratio, *CI* Confidence interval, *BMI* body mass index, *DM* diabetes mellitus, *CABG* coronary artery bypass grafting

The results of the subgroup analysis for the primary outcome are shown in Fig. 3. The associations between the TyG index and MACEs were generally consistent across the subgroups. We did not observe a significant interaction between the TyG index and age, sex, BMI, hypertension or hyperlipidemia (all *p* values for interaction ≥ 0.120). Although no interaction was found between hyperlipidemia and the TyG index, statistical significance was observed only among patients without hyperlipidemia (Fig. 3).

### The incremental predictive value of the TyG index

**Fig. 4:**
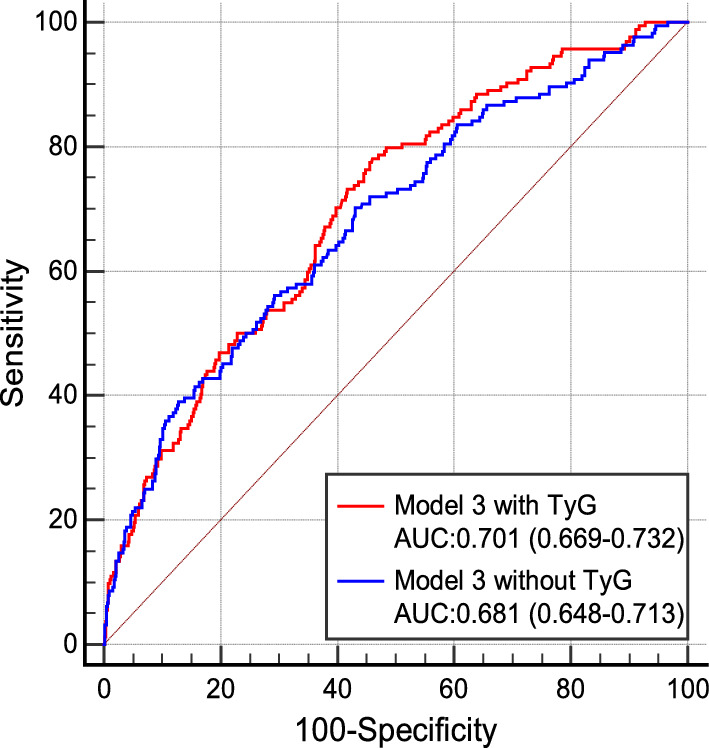
ROC curves for the prediction of MACE. *ROC curve* receiver operating characteristic curve, *TyG index* triglyceride-glucose index, *MACE* major adverse cardiovascular events, *AUC* area under the receiver operating characteristic curves

We assessed the discrimination and reclassification of Model 3 with and without the TyG index for the prediction of MACEs. Compared with Model 3 without the TyG index [area under the receiver operating characteristic curve (AUC) = 0.681], the AUC reached 0.701 when the TyG index was included in Model 3 at the end of the full follow-up (Fig. 4). The addition of the TyG index yielded a significant improvement in the C-statistic, NRI and IDI. In addition, the nonevent NRI, rather than the event NRI, was statistically significant, indicating that the addition of the TyG index could improve the specificity of the model without sacrificing sensitivity (Table 5).

**Table 5:** The incremental prognostic ability of the TyG index

	Model 3 without TyG index	Model 3 with TyG index	*p*-value
C-Statistic (95%CI)	0.656 (0.611–0.701)	0.680 (0.639–0.721)	**< 0.001**
Continuous NRI (95%CI)	Reference	0.269 (0.100–0.438)	**0.002**
Event NRI (95%CI)	Reference	0.098 (− 0.055–0.250)	0.209
Non-event NRI (95%CI)	Reference	0.171 (0.096–0.246)	**< 0.001**
IDI (95%CI)	Reference	0.014 (0.003–0.025)	**0.014**

*NRI* net reclassification improvement, *IDI* integrated discrimination improvement

*p* values in bold are < 0.05

## Discussion

Our study investigated the prognostic ability and clinical utility of the TyG index for nondiabetic patients after CABG. Our current research found that the TyG index may be an independent predictor for post-CABG MACEs driven by stroke and all-cause mortality, and the relationships of the TyG index with adverse events were generally consistent across subgroups. Moreover, the global performance (both risk discrimination and reclassification) of the baseline model may be improved by the addition of the TyG index.

Insulin resistance (IR) is a general term used to describe impaired insulin-mediated glucose uptake in adipose tissue, skeletal muscle, liver and pancreas, and has been regarded as a predictor for adverse outcomes in patients after myocardial revascularization [29, 30]. The hyperinsulinemic-euglycemic clamp test and homeostasis model assessment of IR (HOMA-IR) were used to assess IR. However, there are limitations in conventional assessment methods [31–33]. The calculation of the TyG index was easier than conventional methods and its reliability has been proven in previous studies [14, 34]. Recent studies have revealed that the TyG index is associated with adverse events in patients after PCI [35–38]. Chen et al. and Zhang et al. also found that the TyG index may be an effective indicator of worse prognosis in patients with DM who underwent CABG [23, 24].

Previous studies have shown that the effect of IR on adverse events after CABG was stronger in non-DM patients than in DM patients [11]. For patients who have developed DM, the leading risk factors of MACEs were traditional factors instead of insulin resistance [39]. In addition, various hypoglycemic drugs taken by participants with DM could influence the level of glucose, thereby influencing the calculation of the TyG index. Therefore, it is meaningful to explore the association between the TyG index and adverse events in nondiabetic patients.

In the current study, we demonstrated for the first time the predictive value of the TyG index for MACEs after CABG in patients without DM. IR occurs many years before type 2 DM onset and contributes to the elevated risk of cardiovascular disease and its complications [40]. Our findings showed that the TyG index could be used for risk stratification in nondiabetic patients after CABG and guide early intervention. One thing to note is that the difference in MACE rates among patients with different TyG levels was primarily driven by all-cause death and stroke, instead of nonfatal MI or revascularization. The predictive value of the TyG index for MI and revascularization needs further research. Moreover, nondiabetic patients may have different metabolism profiles. The potential mechanisms that contribute to the predictive role of the TyG index for adverse events in nondiabetic patients still need further investigation.

In our present study, the results were robust in the sensitivity analysis. After excluding noncardiac death, the relationship between the TyG index and MACEs persisted. Lipid-lowering treatment could affect lipid levels and further influence the TyG index. The exclusion of participants taking lipid-lowering drugs at admission did not affect our results. In addition, our findings revealed for the first time the prognostic value of the TyG index in different subgroups of patients after CABG. This association seems to be more prominent in patients without hyperlipidemia. This may be due to various medications taken by participants with hyperlipidemia, which could influence the levels of lipids and glucose, thereby influencing the calculation of the TyG index.

Improved outcome prediction by the TyG index has been proven by several previous studies [21, 22, 35], whereas the usefulness of the TyG index in the improvement of MACE prediction was uncertain in patients after CABG. In the present study, we found that adding the TyG index to the baseline model provided a statistically significant improvement in risk discrimination and reclassification. However, when we further divided the NRI into “event NRI” and “nonevent NRI”, we found that the overall NRI was driven by the nonevent NRI, indicating that adding the TyG index into the baseline model may not be very useful in predicting a greater number of events. Whether the addition of the TyG index can improve the sensitivity of the model needs further research.

This study has several limitations that merit discussion. First, this study is a retrospective observational study with a relatively small sample size and a lack of a control group. Second, insulin levels were not routinely measured in these patients, which makes it impossible to compare the predictive values of HOMA-IR and the TyG index. Third, HbA1c was not measured in most patients and there may have been patients with undiagnosed DM in the cohort. In addition, we only excluded individuals with self-reported new-onset DM in the sensitivity analysis. We cannot completely exclude the interference of undiagnosed DM and newly developed DM. Fourth, non-inclusion of the severity of CAD made the baseline model weak, and the improvement of the C-statistics may be partially attributed to the inadequate adjustment of the baseline model. Finally, the TyG index was evaluated only once at admission. There may be a measurement error, and we are unable to determine the association between the cumulative TyG index and the risk of adverse events. Further prospective studies with comprehensive laboratory evaluations and multiple longitudinal measurements are needed to confirm and extend our findings.

## Conclusion

In conclusion, our data demonstrate that the TyG index was a valuable predictor of MACEs in nondiabetic patients after CABG, and the prognostic value was more prominent among patients without hyperlipidemia. Meanwhile, the addition of the TyG index could improve the predictive performance of the baseline model. Taken together, the TyG index may be a useful marker for risk stratification and outcome prediction in nondiabetic patients after CABG.

### Supplementary Information

**Additional file 1: Table S1.** Sensitivity analysis for the association between the TyG index and MACE.

## Data Availability

The datasets used and/or analyzed during the current study are available from the corresponding author on reasonable request.

## Abbreviations

TyG index: Triglyceride-glucose index
IR: Insulin resistance
CABG: Coronary artery bypass grafting
MACE: Major adverse cardiovascular events
NRI: Net reclassification improvement
IDI: Integrated discrimination improvement
CAD: Coronary artery disease
DM: Diabetes mellitus
PCI: Percutaneous coronary intervention
ACS: Acute coronary syndrome
LVEF: Left ventricular ejection fraction
FH-CAD: Family history of coronary artery disease
FPG: Fasting plasma glucose
TC: Total cholesterol
TG: Triglyceride
LDL-C: Low-density lipoprotein cholesterol
HDL-C: High-density lipoprotein cholesterol
SCr: Serum creatinine
BMI: Body mass index
MI: Myocardial infarction
eGFR: Estimated glomerular filtration rate
SD: Standard deviation
OPCABG: Off-pump coronary artery bypass grafting
VIF: Variance inflation factor
ROC curve: Receiver operating characteristic curve
AUC: Area under the receiver operating characteristic curve
HOMA-IR: Homeostasis model assessment for insulin resistance
ACEI: Angiotensin-converting enzyme inhibitors
ARB: Angiotensin receptor blockers
EuroSCORE: European System for Cardiac Operative Risk Evaluation score
IABP: Intra-aortic balloon pump

## References

[1] Virani SS, Alonso A, Benjamin EJ (2020). Heart disease and stroke statistics-2020 update: a report from the American Heart Association. Circulation.

[2] Vos T, Lim SS, Abbafati C, Abbas KM, Abbasi M, Abbasifard M (2020). Global burden of 369 diseases and injuries in 204 countries and territories, 1990–2019: a systematic analysis for the Global Burden of Disease Study 2019. Lancet.

[3] Neumann FJ, Sousa-Uva M, Ahlsson A (2019). 2018 ESC/EACTS guidelines on myocardial revascularization. Eur Heart J.

[4] Alexander JH, Smith PK (2016). Coronary-artery bypass grafting. N Engl J Med.

[5] Giustino G, Serruys PW, Sabik JF, Mehran R, Maehara A, Puskas JD (2020). Mortality after repeat revascularization following PCI or CABG for left main disease: the EXCEL trial. JACC Cardiovasc Interv.

[6] Creager MA, Lüscher TF, Cosentino F, Beckman JA (2003). Diabetes and vascular disease: pathophysiology, clinical consequences, and medical therapy: part I. Circulation.

[7] Bornfeldt KE, Tabas I (2011). Insulin resistance, hyperglycemia, and atherosclerosis. Cell Metab.

[8] Beverly JK, Budoff MJ (2020). Atherosclerosis: pathophysiology of insulin resistance, hyperglycemia, hyperlipidemia, and inflammation. J Diabetes.

[9] Kogan A, Ram E, Levin S, Fisman EZ, Tenenbaum A, Raanani E (2018). Impact of type 2 diabetes mellitus on short- and long-term mortality after coronary artery bypass surgery. Cardiovasc Diabetol.

[10] Angeloni E, Melina G, Benedetto U, Refice S, Capuano F, Roscitano A (2012). Metabolic syndrome affects midterm outcome after coronary artery bypass grafting. Ann Thorac Surg.

[11] Kajimoto K, Kasai T, Miyauchi K, Hirose H, Yanagisawa N, Yamamoto T (2008). Metabolic syndrome predicts 10-year mortality in non-diabetic patients following coronary artery bypass surgery. Circ J.

[12] Abbasi F, Reaven GM (2011). Comparison of two methods using plasma triglyceride concentration as a surrogate estimate of insulin action in nondiabetic subjects: triglycerides × glucose versus triglyceride/high-density lipoprotein cholesterol. Metabolism.

[13] Simental-Mendía LE, Rodríguez-Morán M, Guerrero-Romero F (2008). The product of fasting glucose and triglycerides as surrogate for identifying insulin resistance in apparently healthy subjects. Metab Syndr Relat Disord.

[14] Vasques AC, Novaes FS, de Oliveira MS, Souza JR, Yamanaka A, Pareja JC (2011). TyG index performs better than HOMA in a Brazilian population: a hyperglycemic clamp validated study. Diabetes Res Clin Pract.

[15] Li X, Sun M, Yang Y, Yao N, Yan S, Wang L (2022). Predictive effect of triglyceride glucose-related parameters, obesity indices, and lipid ratios for diabetes in a Chinese population: a prospective cohort study. Front Endocrinol (Lausanne).

[16] Jian S, Su-Mei N, Xue C, Jie Z, Xue-Sen W (2017). Association and interaction between triglyceride-glucose index and obesity on risk of hypertension in middle-aged and elderly adults. Clin Exp Hypertens.

[17] Son DH, Lee HS, Lee YJ, Lee JH, Han JH (2022). Comparison of triglyceride-glucose index and HOMA-IR for predicting prevalence and incidence of metabolic syndrome. Nutr Metab Cardiovasc Dis.

[18] Lee SB, Ahn CW, Lee BK, Kang S, Nam JS, You JH (2018). Association between triglyceride glucose index and arterial stiffness in Korean adults. Cardiovasc Diabetol.

[19] Park K, Ahn CW, Lee SB, Kang S, Nam JS, Lee BK (2019). Elevated TyG index predicts progression of coronary artery calcification. Diabetes Care.

[20] Won KB, Park EJ, Han D, Lee JH, Choi SY, Chun EJ (2020). Triglyceride glucose index is an independent predictor for the progression of coronary artery calcification in the absence of heavy coronary artery calcification at baseline. Cardiovasc Diabetol.

[21] Jiao Y, Su Y, Shen J, Hou X, Li Y, Wang J (2022). Evaluation of the long-term prognostic ability of triglyceride-glucose index for elderly acute coronary syndrome patients: a cohort study. Cardiovasc Diabetol.

[22] Wu Z, Liu L, Wang W, Cui H, Zhang Y, Xu J (2022). Triglyceride-glucose index in the prediction of adverse cardiovascular events in patients with premature coronary artery disease: a retrospective cohort study. Cardiovasc Diabetol.

[23] Chen L, Ding XH, Fan KJ, Gao MX, Yu WY, Liu HL (2022). Association Between triglyceride-glucose index and 2-year adverse cardiovascular and cerebrovascular events in patients with type 2 diabetes mellitus who underwent off-pump coronary artery bypass grafting. Diabetes Metab Syndr Obes.

[24] Zhang H, Chong H, Li Z, Li K, Zhang B, Xue Y (2022). Triglyceride-glucose index in the prediction of major adverse cardiovascular events in patients with type 2 diabetes mellitus after coronary artery bypass surgery: a retrospective cohort study. Front Endocrinol (Lausanne).

[25] Cutlip DE, Windecker S, Mehran R, Boam A, Cohen DJ, van Es GA (2007). Clinical end points in coronary stent trials: a case for standardized definitions. Circulation.

[26] Hong S, Han K, Park CY (2020). The triglyceride glucose index is a simple and low-cost marker associated with atherosclerotic cardiovascular disease: a population-based study. BMC Med.

[27] Levey AS, Stevens LA, Schmid CH, Zhang YL, Castro AF, Feldman HI, Kusek JW, Eggers P, Van Lente F, Greene T (2009). A new equation to estimate glomerular filtration rate. Ann Intern Med.

[28] Guerrero-Romero F, Simental-Mendía LE, González-Ortiz M, Martínez-Abundis E, Ramos-Zavala MG, Hernández-González SO (2010). The product of triglycerides and glucose, a simple measure of insulin sensitivity. Comparison with the euglycemic-hyperinsulinemic clamp. J Clin Endocrinol Metab.

[29] Nishimura M, Tokoro T, Nishida M, Hashimoto T, Kobayashi H, Yamazaki S (2008). Association of insulin resistance with de novo coronary stenosis after percutaneous coronary artery intervention in hemodialysis patients. Nephron Clin Pract.

[30] Uetani T, Amano T, Harada K, Kitagawa K, Kunimura A, Shimbo Y (2012). Impact of insulin resistance on post-procedural myocardial injury and clinical outcomes in patients who underwent elective coronary interventions with drug-eluting stents. JACC Cardiovasc Interv.

[31] Pacini G, Mari A (2003). Methods for clinical assessment of insulin sensitivity and beta-cell function. Best Pract Res Clin Endocrinol Metab.

[32] Rudvik A, Månsson M (2018). Evaluation of surrogate measures of insulin sensitivity—correlation with gold standard is not enough. BMC Med Res Methodol.

[33] Manley SE, Stratton IM, Clark PM, Luzio SD (2007). Comparison of 11 human insulin assays: implications for clinical investigation and research. Clin Chem.

[34] Luo P, Cao Y, Li P, Li W, Song Z, Fu Z (2022). TyG index performs better than HOMA-IR in Chinese type 2 diabetes mellitus with a BMI < 35 kg/m(2): a hyperglycemic clamp validated study. Medicina (Kaunas).

[35] Zhu Y, Liu K, Chen M, Liu Y, Gao A, Hu C (2021). Triglyceride-glucose index is associated with in-stent restenosis in patients with acute coronary syndrome after percutaneous coronary intervention with drug-eluting stents. Cardiovasc Diabetol.

[36] Zhao Q, Zhang TY, Cheng YJ, Ma Y, Xu YK, Yang JQ (2020). Impacts of triglyceride-glucose index on prognosis of patients with type 2 diabetes mellitus and non-ST-segment elevation acute coronary syndrome: results from an observational cohort study in China. Cardiovasc Diabetol.

[37] Hu C, Zhang J, Liu J, Liu Y, Gao A, Zhu Y (2020). Discordance between the triglyceride glucose index and fasting plasma glucose or HbA1C in patients with acute coronary syndrome undergoing percutaneous coronary intervention predicts cardiovascular events: a cohort study from China. Cardiovasc Diabetol.

[38] Luo E, Wang D, Yan G, Qiao Y, Liu B, Hou J (2019). High triglyceride-glucose index is associated with poor prognosis in patients with acute ST-elevation myocardial infarction after percutaneous coronary intervention. Cardiovasc Diabetol.

[39] Laakso M, Kuusisto J (2014). Insulin resistance and hyperglycaemia in cardiovascular disease development. Nat Rev Endocrinol.

[40] Laakso M (2010). Cardiovascular disease in type 2 diabetes from population to man to mechanisms: the Kelly West Award Lecture 2008. Diabetes Care.

