# Supplementary material for: Triglyceride-glucose index in the prediction of adverse cardiovascular events in patients without diabetes mellitus after coronary artery bypass grafting: a multicenter retrospective cohort study
Source: Cardiovasc Diabetol. 2023 Aug 30;22:230. doi: 10.1186/s12933-023-01969-3 (PMC10470170; doi:10.1186/s12933-023-01969-3)
Supplement: Supplementary file 1 — Additional file 1: Table S1. Sensitivity analysis for the association between the TyG index and MACE. [file 12933_2023_1969_MOESM1_ESM.docx]

**Table S1 Sensitivity analysis for the association between the TyG index and MACE**

| **TyG index** | **HR (95% CI)** | | |
| --- | --- | --- | --- |
|  | **Analysis 1** | **Analysis 2** | **Analysis 3** |
| Per Unit increase | 1.94 (1.40-2.70)*** | 1.99 (1.40-2.82)*** | 1.91 (1.40-2.61)*** |
| Per SD increase | 1.42 (1.19-1.69)*** | 1.44 (1.20-1.73)*** | 1.41 (1.19-1.66)*** |
| Tertile 1 | 1 (Reference) | 1 (Reference) | 1 (Reference) |
| Tertile 2 | 1.14 (0.70-1.87) | 1.18 (0.70-1.97) | 1.17 (0.73-1.88) |
| Tertile 3 | 2.33 (1.48-3.65)*** | 2.22 (1.39-3.54)** | 2.38 (1.54-3.66)*** |
| *p* for trend | **< 0.001** | **< 0.001** | **< 0.001** |

Analysis 1: Excluding noncardiac death (n = 20).

Analysis 2: Excluding patients with a history of lipid-lowering usage (n = 174).

Analysis 3: Excluding patients who developed DM during the follow-up (n = 37).

Model adjusted for age, gender, BMI, LVEF, previous MI, previous stroke, previous PCI, left main disease, multivessel disease, current smoking, current drinking, FH-CAD, hypertension, hyperlipidemia, duration of surgery, OPCABG, number of grafts, use of arterial grafts, TC, LDL-C, HDL-C, eGFR, EuroSCORE II, antiplatelet drugs and statins.

HR, Hazard ratio; CI, Confidence interval; SD, standard deviation.

** *p* < 0.01

*** *p* < 0.001

*p* values in bold are < 0.05.
